# Children's Country Holidays Fund

**Published:** 1889-09-21

**Authors:** 


### Children's Country Holidays Fund.

It is particularly pleasant just now to visit the London schools. Those who have worked on the Children's Country Holidays Fund are beginning to reap their reward, for in every class are browner faces and brighter eyes than seemed possible in July. Of course, even if 20,000 children get the benefit of the country fortnight (and the council hope to send that number this year), the proportion in each school still remains small.

The white faces of some who have been left behind look still whiter against those of their more fortunate companions. A few will still go during September through the fund, and the hopping season has taken a few more of the poorest and most ragged for a country change which will be for more than a fortnight. Those who have come back ar full of strange travellers' tales: what the animals do, and what they have seen and heard, becomes a never-failing source of chatter. Real cricket fields; creatures to watch, butterflies to catch, flowers to pick, fresh air, fresh food, fewer scoldings—all these combined make a sum of delights to be long remembered. It is very touching to hear these city-bred children, to whom all these sights and sounds have been so wonderful, dwell with minute emphasis on all the commonplace incidents of country life with which they have for the first time become acquainted. It is easy to see what a tonic for mind as well as body is the fortnight's stay in a country cottage.

